# Supplementary material for: A Separated Receptor/Transducer Scheme as Strategy to Enhance the Gas Sensing Performance Using Hematite–Carbon Nanotube Composite
Source: Sensors (Basel). 2019 Sep 11;19(18):3915. doi: 10.3390/s19183915 (PMC6767046; doi:10.3390/s19183915)
Supplement: Supplementary file 1 [file sensors-19-03915-s001.pdf]

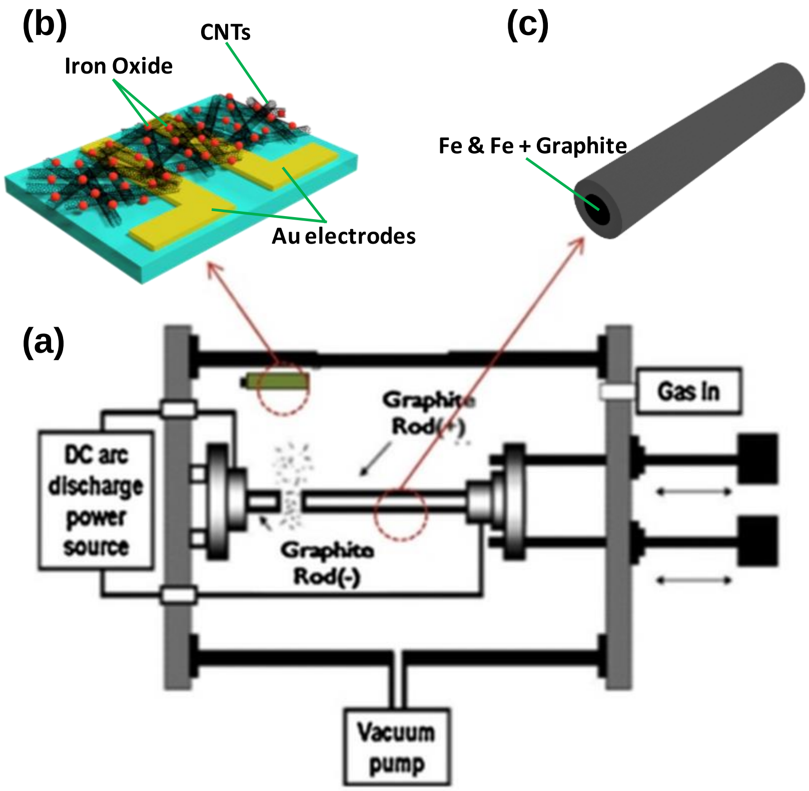
(b)
CNTs
Iron Oxide
Au electrodes
(c)
Fe & Fe + Graphite
(a)
Graphite
Rod(+)
Gas In
DC arc
discharge
power
source
Graphite
Rod(-)
Vacuum
pump

Figure S1. (a) Schematic diagram of the arc-discharge system. (b) The schematic sensor device structure deposited with single-wall carbon nanotubes (SWCNTs) dispersed with iron oxides on Au-electrode patterned alumina substrate. The substrate was mounted on the inside wall of the chamber for the iron (Fe):CNT composite deposition. (c) The graphite tube used as the arc-discharging source. The hollow tube was filled by the controlled mixture of Fe and graphite powder. [1].

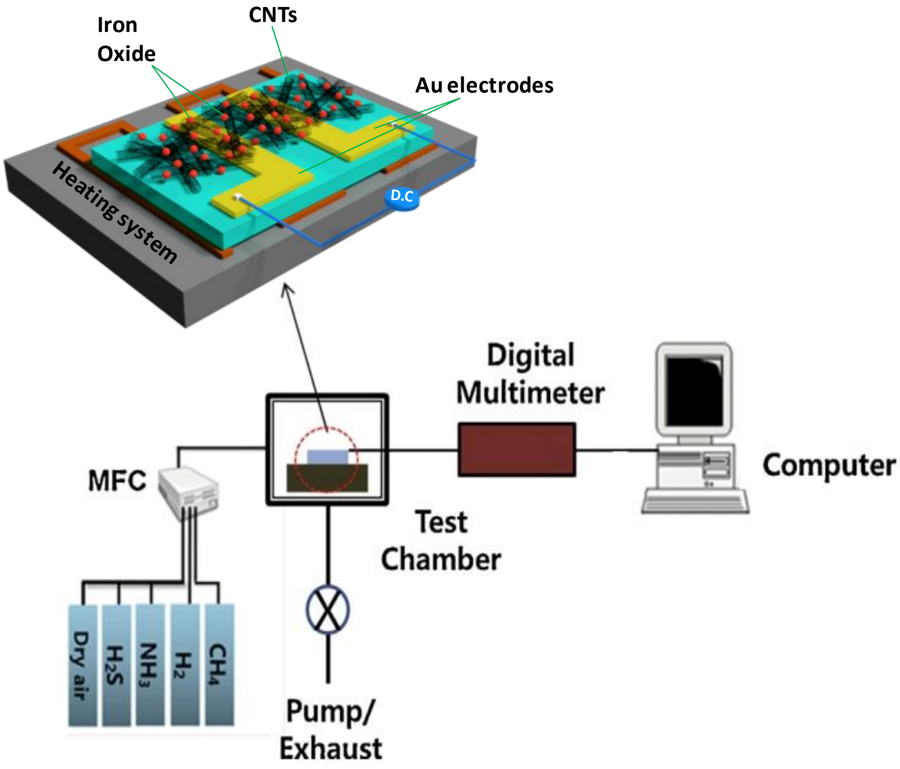

Figure S2. Schematic diagram of the sensor measurement system with the magnified sensor device configuration.[1].

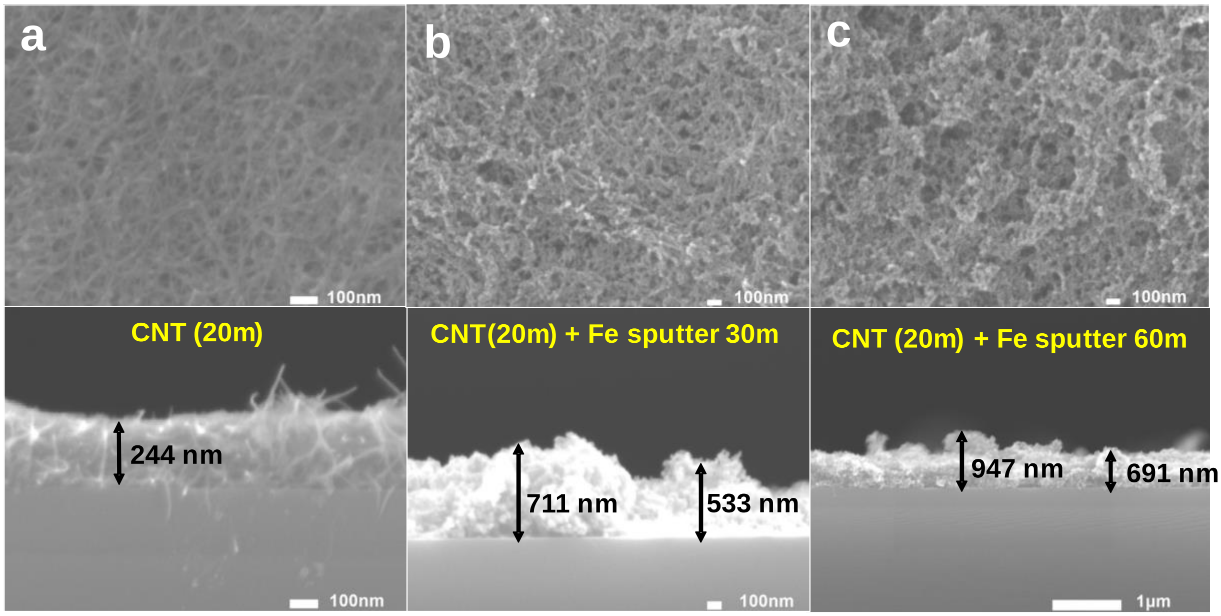

Figure S3. (a) SEM images of the SWCNT mat fabricated by the arc-discharge for 20 min followed by the methanol treatment. (b) Hematite (Fe_2O_3):CNT composite structures fabricated by the sputter deposition of Fe on the SWCNT mat for 30 min and (c) 60 min followed by oxidation at 400 °C for 2 h.

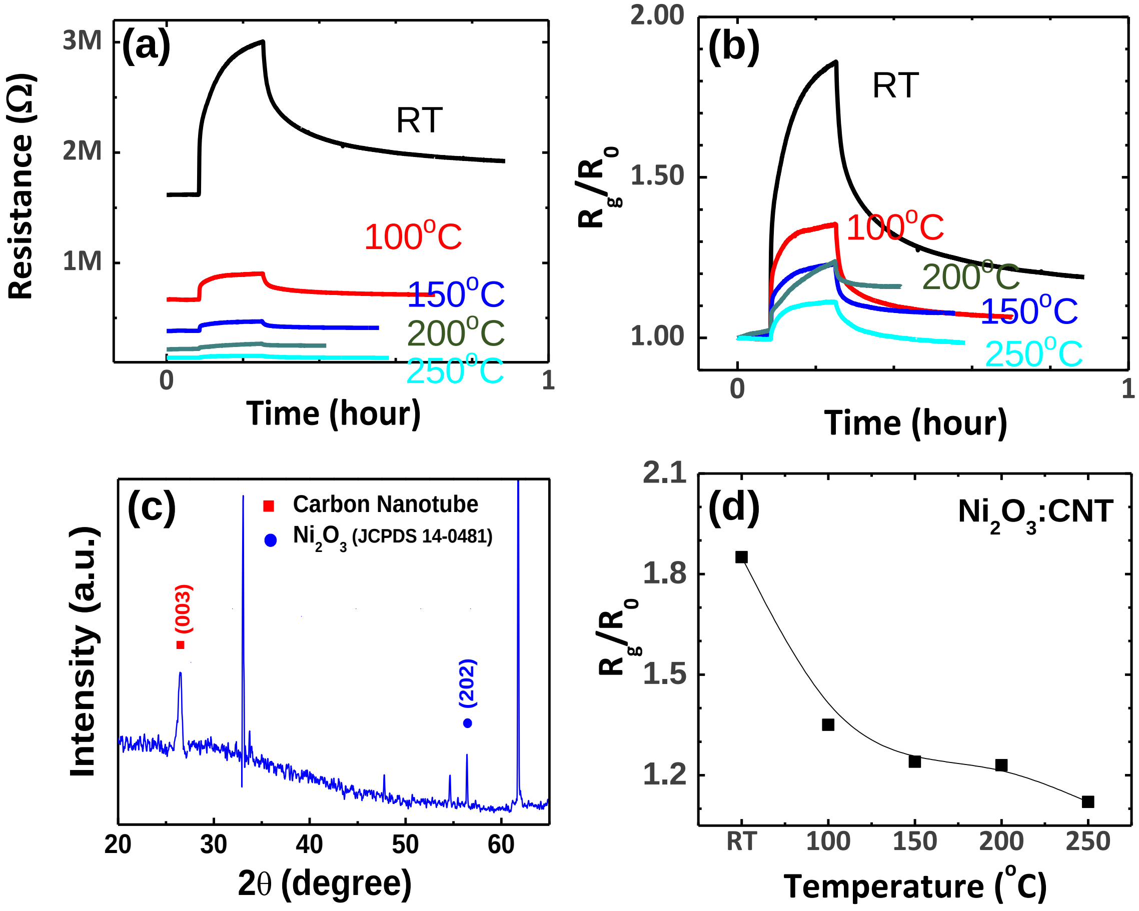

Figure S4. (a, b) Temperature dependence (room temperature (RT) to 250 °C) of nickel-oxide composite structures (Ni_2O_3:SWCNT) to 100 ppm NH_3. (c) XRD pattern of the composite structure. (d) The temperature dependence of ammonia sensing of the Ni_2O_3:CNT composite sensor structure. The result supports that the synergy effect is not from the junction property but is a general result from the separated receptor-transducer scheme.

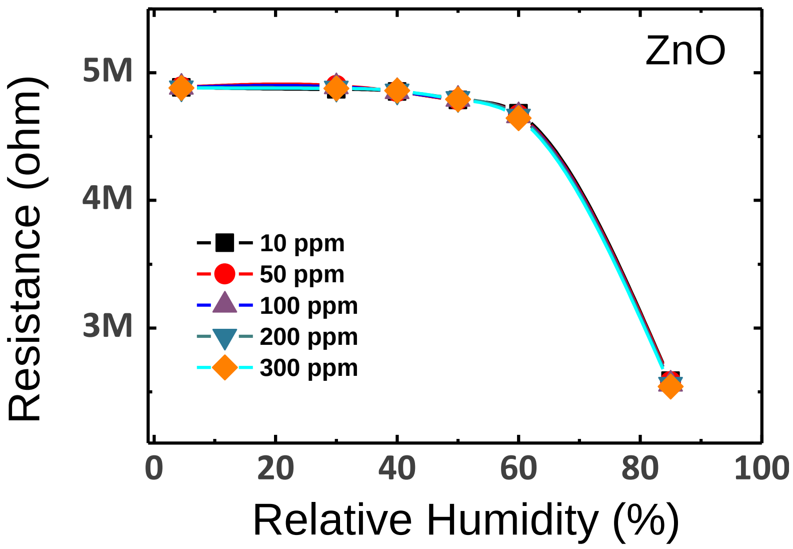

Figure S5. Response of ZnO thin film to varying NH_3 concentration and relative humidity.

References

1. Moon, S., et al., *Co3O4-SWCNT composites for H2S gas sensor application.* Sensors and Actuators B-Chemical, 2016. **222**: p. 166-172.
